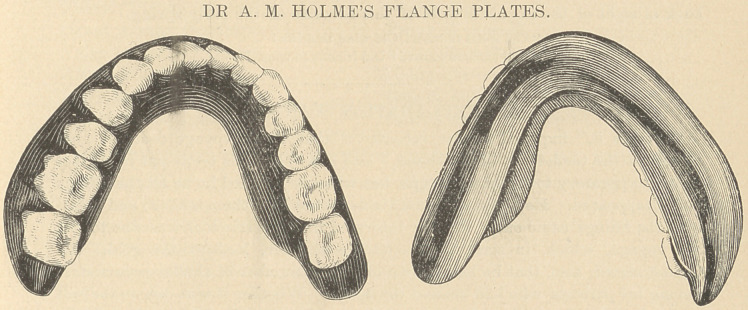# Current News and Opinion

**Published:** 1888-05

**Authors:** 


### Current News and Opinion.

CORRESPONDENCE.

*Editor Independent Practitioner* :—

I must say that I read that part of the report of the International Congress which was published in the March number of your journal, with a great deal of surprise. I cannot comprehend why an editor who is usually so fearless in attacking that which he believes to be false practice should allow such matter to be inserted without comment.

Dr. Cunningham, of England, especially, preaches some doctrines which I have always been taught to believe, and which I am fully convinced, are about as bad practice as can well be conceived. He says that "his practice with pulpless teeth is to remove the soft dentine, clearing out the pulp-chamber, but not the root canals. When the cavity is ready for filling, he places over the entrance to the canals a disk of paper saturated with creosote in which ten or fifteen grains of arsenic has been dissolved. Over this he fills with oxy-chloride, and finishes the filling as desired."

He says that he usually fills all classes of teeth at one sitting, rarely seeing the patient again. Now if I should follow such a practice, I not only should not expect to see the patient a second time, but I should pray that I might not. If the leaving of a canal uncleansed and unfilled, with an arsenic preparation at its opening, is good practice, then certainly I have not been well taught, and if Dr. Cunningham's patients do not eventually curse the day when they placed themselves under his care, then there is no truth in pathology as usually taught in this country. If it be no matter whether or not broken drills be left in the tooth, and if dead teeth can be successfully treated and filled in half an hour, whence the necessity for spending three years in study? Such practice as that may be learned in a week. Indeed, it seems to come naturally to some dentists.

There were others who took part in that debate, some of whose utterances were, in my opinion, nearly as ill-advised and erroneous, and I cannot comprehend how you and others who were there allowed these things to pass without rebuke.

H. A. Birdsall, D. D. S.

The editor of this journal was not present at the final session, as he found that by the route over which he must return home no train left Washington between early Friday evening and Monday. Besides, he had already expressed himself, perhaps more forcibly than was necessary, and certainly much more so than he realized until he saw his remarks in print, upon this very question of root treatment. It was but fair that members who held views differing from those usually accepted should have their innings in turn, and that their opinions should receive respectful treatment at the hands of the reporters —Editor.

---

### THE AMENDMENT TO THE DENTAL ACT.

The following bill has passed both branches of the Legislature of the State of New York. It will be seen that some of its provisions are of great importance. The bill has been considerably amended since its first introduction.

### AN ACT

To amend chapter five hundred and forty of the laws of eighteen hundred and seventy-nine, entitled "An act to regulate the practice of dentistry in the State of New York."

*The People of the State of New York, represented in Senate and Assembly, do enact as follows:*

Section 1. Section one of chapter five hundred and forty of the laws of eighteen hundred and seventy-nine, entitled "An act to regulate the practice of dentistry in the State of New York," is hereby amended so as to read as follows:

Sec. 1. It shall be unlawful for any person to practice dentistry in the State of New York for fee or reward or to assist in the practice of operative dentistry as either agent or employee, unless he shall have received a proper diploma or certificate of qualification from the State Dental Society or from the faculty of a reputable dental or medical college, recognized as such by said society, and shall be duly registered and shall have received a certificate thereof, as provided in section three of this act; provided that persons who were engaged in the practice of dentistry in the State of New York on the twentieth day of June, eighteen hundred and seventy-nine, who shall comply with the requirements of section three of this act, shall be otherwise exempt from the provisions of this section, and provided further that nothing contained in this section shall prevent a student who is pursuing a regular course of instruction from assisting a person in the practice of dentistry qualified as hereinbefore provided.

SEC. 2. Section three of said act is hereby amended so as to read as follows:

SEC. 3. Every person practicing dentistry within this State shall register in the office of the clerk of the county where his place of business is located, and in the office of the clerk of any county into which he shall remove his place of business, in a book to be prepared and kept by the clerk for that purpose, giving his name, office and postoffice address and the date of such registration, and shall, on presenting to the county clerk a certificate from the member of the State Board of Censors appointed by the State Dental Society for the judicial district in which such county is situated that he has received a proper diploma or certificate of qualification as provided in section one of this act, be entitled to register and receive a certificate of such registration upon payment to the clerk of a fee of fifty cents.

---

TRADE OR PROFESSION?

We have before us an address delivered before the Maine Dental Society, by Horatio C. Merriam, D. M. D., in which is discussed the question whether dentistry is a specialty of medicine or whether it is a trade. The distinction between trade and profession is presented in a manner which deserves a larger number of readers than is comprised in strictly dental circles

What would be thought of Dr. Bigelow, the address asks, and how would his name go down in the history of medicine if he had asked or received from his brothers a royalty for each time they had performed his operation for stone, or sold his instruments so that they could be withdrawn from competing with those already in the market? Or even patented and received a revenue on their sale? Would a dealer even venture to approach such a man with a proposition to buy his instruments so as to control their sale? Yet these things are regarded as legitimate and proper in trade, but they are condemned in medicine, for the reason that methods honorable in trade may yet be discreditable in a liberal profession The condition of the general medical profession as shown by such an example differs widely from the position of the dentists, and the author gives various examples of an opposite course of conduct. "A large house has acquired control of all patents on the dental engine, and is thus at liberty to manufacture such only as it may wish, and place its own judgment instead of the profession's." "A dental chair, the invention of a dentist, had been bought and withheld from the profession." "I heard within a few months of a handpiece that a manufacturer had bought for five years, and had thus for five years prevented its coming into competition with those of his own make" "A short time ago I was told of a dentist who took to a manufacturer a form of tooth he had invented. The manufacturer looked at it, then opened a drawer and showed by drawings that he already had the invention in his possession." "Many of our journals are published and controlled by dealers, and often contain articles in the text advertising materials for sale by their publishers. We often see recommended or offered for sale to the profession articles and medicines, the formulas of which are not given "

"Our dealers have also formed a combination and propose to decide who shall conduct our supply-business, whose gold they shall sell you, whose material they will or will not keep, through what firms you shall order their goods, and they are able to take orders for only such goods as the combination chooses to offer. They may have the power to interfere with the delivery of an instrument you have ordered if it infringes on a patent held by them."

The author then goes on to say that "they are perfectly right in all that they do or have done if dentistry is a trade and we are tooth carpenters . . . The brother who sells his invention instead of bringing it before his Society . . . is the one to blame, not the dealer who buys it and shelves it to his advantage. Surgical instrument makers would soon learn to do this if the medical profession would permit." The fact that the onus of such a condition is the fault of the profession and not of the dealers, is enforced by the question; What would be thought of a Cheever, or a Hodges, or any leading surgeon who would do such a thing as patent and sell his inventions? Yet it would be right if medicine were a trade and they repairers of the clavical and menders of femurs.

Now these extracts present so forcibly the distinction between a trade and a profession that they may well be pondered by the medical profession at large. If at times it seems a little unfair that one's improvements on surgical apparatus should not directly bring in a fixed income, the opposite condition, when everybody should exact royalties and conceal the composition of all new drugs, is too horrible to contemplate—and between perfect freedom on the one hand and patents and copyrights on all new inventions on the other, there can be no middle ground. It is certainly entertaining and instructive to contemplate the unpleasant position which might be, were the general profession suddenly to overturn its present lofty ideal and become a trade.

It would be pleasant to believe the statement as to the position of the dentists an exaggerated one, but as a warning it is perhaps not useless nor entirely needless.

NOTE.—The above editorial from *The Boston Medical and Surgical Journal* is given a place at the request of one of the most respected members of the dental profession.—EDITOR.

---

### AN INTERNATIONAL DENTAL CONGRESS.

The *Dental Review*, of March 15, 1888, in advocating the holding of "An International Dental Congress at Paris, France, in September, 1889," disclaims any intention of interfering with a Section of Dental and Oral Surgery in connection with the Tenth International Medical Congress to be held in Berlin, 1890. Notwithstanding this disclaimer, it is difficult to see how the editor of the *Review* could more directly and certainly interfere with the organization of an efficient and successful Dental Section of the International Medical Congress in Berlin, than by persisting in his scheme of forestalling it by a separate International Dental Congress the year preceding in Paris. The full recognition of properly educated dentists by the successful organization of a Section of Dental and Oral Surgery, as a part of the great International Medical Congress at London, in 1881, and i s repetition with still greater success as a part of the International Medical Congress at Washington. in 1887, leaves no room for doubt about the purpose of organizing a similar Section in the next Congress at Berlin, and of its permanent recognition as a legitimate department of the great field of medicine and surgery. Then why should not every enlightened member of the profession use his influence for perfecting the unity of all the departments, and the promotion of such harmony in the organization as will afford mutual support and mutual advancement. There is no interest, social, scientific or practical, to be promoted by an exclusive International Dental Congress in Paris next year, that could not be more efficiently promoted by a Section of the International Medical Congress the following year at Berlin. The published proceedings of a Congress of Dentists will reach but few outside of its own members, while the work of a Section becomes a part of the published transactions of the general Congress, and thus receives a wide di-tribution to members of all other Sections, and *vice versa*, the work of all other Sections becomes the property of the members of the Dental Section. So true it is, that co-operation and union impart strength and diffuse knowledge, while segregation and exclusiveness limit both.—*Jour. Am. Med. Ass'n.*

### DR A. M. HOLME'S FLANGE PLATES.

The above cuts represent a form of plate which is intended for lower cases in which the alveolus is very much absorbed. The cuts are made from a plate which Dr. Holmes says is an exact duplicate of a practical case that has been in use for eight months, and has proved very satisfactory indeed. They do not represent it very well, except upon one side, but it should be comprehended that the flange extends entirely around the inside of the plate. The upper side is concave, and in the concavity the tongue rests and holds the plate in position. The only difficulty in making such a plate is to determine just where to place the flange so that it shall not irritate the genio- and hyoglossal muscles. It should be placed as low as possible. Dr. Holmes says that a plate properly made in this way has been entirely serviceable in a mouth in which every other form had proved a failure, and his experience is that in many cases it will be found superior to any other. It is sometimes extremely useful in partial plates, which can thus be made thinner and lighter than by the usual mode of construction. The method of forming the flange will readily be comprehended from the cuts.

### A BREATH OF SPRING.

The practitioner in a large city who is closely confined to his office work knows no Spring, and has little personal appreciation of the change of seasons save in the mere modification of temperature. Seeing little out of doors besides brick walls and stone pavements, he has no sense of the awakening of a world from the winter's trance, aside from his general participation in the universal uneasiness and unrest, until perchance at an unexpected moment he meets with a new birth of nature, a tender youngling but just entered upon a season's existence, and feels his own inner nature expanding in sympathy with the opening buds.

A well-known dentist who lives in the picturesque regions of Pennsylvania, in memory of old times and of old friends whom we shall meet no more on earth, each year sends us a box of that delicate harbinger of the coming summer, the beautiful Trailing Arbutus. He knows only too well the memories which it awakens, and the tender recollections of the long ago which come thronging back at sight of the exquisite tints of this favorite of the early spring.

"Old wood to burn! Old wine to drink!
Old authors to read! Old friends to trust!"

### AN EXPLANATION WANTED.

"Why is it," inquired a lady, recently, "that a sea voyage destroys gold fillings in the teeth?" "My husband," said she "had his teeth put in perfect order preparatory to a trip to Europe, but when he reached home all the fillings had disappeared. He called again on his dentist to have them refilled, and asked why the fillings came out. He was informed that the mischief was due to the sea voyage, which, in some unaccountable manner, loosened the gold. His dentist stated, also, that he had known of quite a number of similar occurrences among his patients who had crossed the ocean. Whether it was due to the salt atmosphere or the motion of the vessel, he could not say." We wonder which!

F.

### THE THREE HOTTEST DAYS IN 1888.

In his article on "Where to Spend the Summer," in *Scribner's* for April, Gen. Greely, chief signal officer, makes a prophecy as to the hottest days in the year 1888. The Detroit *Journal*, taking the matter up, has offered a prize of $500 to the person guessing correctly, before June 1st, what the three days will be Gen. Greely immediately telegraphed his guess to the paper, in accord with his reasons in *Scribner's* for April

### CHICAGO COLLEGE OF DENTAL SURGERY.

During the College year just closed, one hundred and twenty-six students were matriculated. The commencement exercises occurred Tuesday, March 27th, at 2 30 P. M., at the Grand Opera House. The Faculty address was delivered by Prof. Truman W. Brophy, and the class valedictory by Dr. A H. Peck.

The degree of D. D. S. was conferred upon the following gentlemen:—

John Wesley Alderson,

John Charles Barclay,

George Heinrich Becker,

Clayton William Bennett,

Orrin George Bennett,

Frank William Cady,

Sherman Lee Chappell,

Frank Beaumont Clarke,

Rush Eugene Crissman,

William Gould Dalrymple,

Charles Henry Darling,

Frank Henry Davis,

Samuel Finley Duncan,

William Andrew Fortuin,

Clarence Barnard Freeman,

Robert Curtis Gardner,

Thomas Dimma Gardner,

Grant Arthur Goodrich,

Valentine Arthur Gudex,

Alfred Ward Hebert,

Peter Monroe Hendershott,

Albert Frank Henkel,

Thomas Francis Henry,

Richard Herrmann,

James Ward House,

Henry K Kerman,

Richard Kessel,

William Kuester,

Louis Frank Lattan,

George Edward Long,

Alfred Lowther,

Anthony Mann,

Clare Winchel Marshall,

Edward Martin McIntosh,

Charles James Merriman,

Ewing Van Darian Morris, M. D

Hans Theodore Nordahl,

Adelbert Henry Peck,

George Reedy,

Frank M. Russell,

Harry Reid Staley,

Henry Stewart,

Rupert DeGeorge Treen,

Samuel Adolphus Whedon.

---

### FIRST DISTRICT DENTAL SOCIETY OF THE STATE OF NEW YORK.

At the annual meeting of the above society, held Tuesday evening, April 3, 1888, the following were elected officers for the ensuing year :—

*President*—W. W. Walker,
*Vice-President*—J. F. P. Hodson.
*Secretary*—B. C. Nash.
*Treasurer*—John I. Hart
*Librarian*—J. Bond Littig.
*Board of Censors for Five Years*—A. L. Northrop, Frank Abbott. S. G. Perry, William Carr and A. R. Starr.
*Delegates to the State Dental Society for Four Years*—J. W. Taylor and B. A. R. Ottolengui.

B. C. NASH, Secretary.

---

### PENNSYLVANIA STATE DENTAL SOCIETY.

The twentieth annual meeting of the Pennsylvania State Dental Society will be held in Philadelphia, Pa., Tuesday, June 5th, 1888. Session to continue for three days.

WM. B. MILLER, D. D. S., Rec. Sec'y.

### NATIONAL DENTAL ASSOCIATION, U. S. A.

The National Dental Association of the United States of America will hold its next regular meeting at Washington, D. C., July 24, 25 and 26 1888.

For this meeting, as for all former ones, the authorities of the Smithsonian Institute have kindly granted the use of the Lecture Hall of the U. S. National Museum.

All members of the profession in good standing are invited to be present.

Art. II, Sec. 1 of Constitution.—The future membership of this Association shall be composed of dentists who may be elected upon application, which application shall be accompanied by credentials of membership in a State Society, or by a recommendation from five members of this Association, or of his State Society.

R. Finley Hunt, D. D. S., Sec N. D. A., U. S. A

---

### CHICAGO DENTAL SOCIETY.

At the annual meeting held on Tuesday evening, April 3, 1888, the following named persons were elected officers for the ensuing term :—

*President*—J. A Swasey.

*First Vice-President*—J. W. Wassall.

*Second Vice-President*—W B. Ames.

*Recording Secretary*—C. N. Johnson.

*Corresponding Secretary*—Louis Ottofy.

*Treasurer*—E. D. Swain.

*Librarian*—A. W. Harlan.

*Executive Committee*—Edmund Noyes, Geo. H. Cushing, J. N. Crouse.

Louis Ottofy, Cor. Sec.

---

Dr. E. L. Townsend, of Los Angeles, Cal., says, in the *Southern California Practitioner*, that after quite an extensive examination of the teeth of the Chinese, he feels sure that the statement lately made that there are never any irregularities in the teeth of the Chinese, is based upon anything but actual observation. A regular denture among them is more of a rarity than among the whites. In meeting them upon the street the various irregularities are constantly observed, and upon closer examination all the diseases common to the whites are found. Dr. Townsend has observed all forms of irregularity, and is fully convinced that irregularities of the teeth are as prevalent with the Chinese as with any other race. The editor of the *Southern California Practitioner* confirms Dr. Townsends observations.

---

The Southern Dental Journal in view of its late discipline of pillaging editors (every word of which we heartily approved), should be extremely watchful of its own pages. The last number contains an instance in point in which its own rule is not only broken, but additional injury is inflicted by disguising an extract from an article in this journal by a false head.

We do not mean to imply that this was anything more than carelessness on the part of our usually scrupulous contemporary, but it shows how even great Homer may nod, and that with the best intentions one may err.

THIS SEEMS TO BE the day of tooth matrices. In the advertising pages, cuts of one which is offered to the profession by Dr. C. Stoddard Smith will be found, which is not only effective, but cheap and simple. The band for this device is manufactured for the case in hand, and is cut from Taggers' tin. This matrix has some advantages that are peculiar to it. The band may easily be cut so that it will exactly fit the tooth. If the latter be bell-shaped the band can be cut longer upon one edge than the other, and thus embrace the tooth closely at both cervical and masticating borders. The band being made of a bright reflecting metal, it helps to illuminate the cavity. A single fastening clamp will serve for almost any tooth, and the adaptation can always be depended upon.

---

TO REMOVE IRON RUST.—It is often very difficult, and sometimes impossible, to remove rust from articles made of iron. Those which are most thickly coated are most easily cleaned by being immersed in a nearly saturated solution of chloride of tin. The length of time they remain in this bath is determined by the thickness of the coating of rust. Generally twelve to twenty-four hours is long enough. The solution ought not to contain a great excess of acid, if the iron itself is not to be attacked. On taking them from the bath, the articles are rinsed first in water, then in ammonia and quickly dried. The iron when thus treated has the appearance of dull silver. A simple polishing gives it its normal appearance.—*Popular Science News.*

---

IN FRANCE druggists are not allowed to sell "toxic drugs," which include chloroform and preparations of opium, to dentists, except upon prescription of a physician, health officer or veterinary surgeon. It is not yet decided whether cocaine is to be included in the tabooed list.—*Pharmaceutical Era.*

And so French law places dentists below horse doctors! Is it because the well-being of Frenchmen is of less consequence than that of French cattle, or because French dentists are believed to be less intelligent and well informed than their horse doctors?—EDITOR.

---

DR. W. B. MILLER, of Altöona, Pa., sends us a matrix that in some respects is a decided improvement upon the usual band matrix. The screw which draws it tight is a thumb-screw, and upon the end which bears against the tooth is a safety block or plate which fits against the tooth firmly, and is held in exact position by guide-pins. As a consequence it is easily placed in position, and is not at all liable to slip, while it obviates the danger of fracture of frail walls by pressure. Dr. Miller has also devised a disk case in the form of a cylinder, in which sandpaper disks are held, one being presented for removal at a time. When this is removed a spring pushes the next one into its place. It is very convenient.

---

"PRAY SEND for the best operator for the teeth at Turin where, I suppose, there is some famous one, and let him put yours in perfect order, and then take care to keep them so, afterwards, yourself."—*From "Lord Chesterfield's letters to his son," May* 15, 1749.

PARKE, DAVIS & CO., of Detroit, have perfected a set of hypodermic tablets which will prove extremely useful to all who desire to use aconite, atropine, cocaine, morphine, strychnine and other remedies hypodermically. They are put up in bottles containing twenty-five tablets, each having the proper amount of the remedy for a single dose The tablets instantly dissolve in water, and form a perfectly limpid solution. Parke, Davis & Co. also furnish a hypodermic case containing their latest improved syringe, with points, and six bottles of the tablets in most common use. Full information may be obtained by writing the firm and mentioning this journal.

---

THE FIFTH DISTRICT DENTAL SOCIETY at its annual meeting held in Utica, April 10th and 11th, unanimously voted to request the concurrence of the members of the Sixth, Seventh and Eighth District Societies in a Union Meeting to be held in Syracuse, in October next. The Union Meetings of the Societies named that have been held in the past have been very profitable and pleasant, and there is no room for doubt that the Fifth District will treat its guests with the same consideration that the other Societies have shown.

---

DR. AND EX-SENATOR A. M. HOLMES, of Morrisville, N. Y., sends us a sole-leather disk which is the most effective and perfect polisher for the borders of approximal fillings that we have met with. It is evidently cut from a piece of sole-leather, a hub being left in the centre to give the mandrel point a good hold, but how it is made or whether the Senator will furnish them to others we have not as yet been able to find out. He has, he says, been using them for a number of years.

---

THE DENTAL SOCIETY OF THE STATE OF NEW YORK will meet in Albany, on Wednesday and Thursday, May 9th and 10th.

As the Society does not send out the usual advance notices for publication in the journals, and as the programme was not received until the forms for our May number were made up, it is impossible to present more than this notice, but a good meeting may be expected.

---

TINCTURE OF IRON when diluted with water has a very corrosive action on the teeth, owing to the free acid it contains. This should be neutralized by using for the diluent an alkaline mineral water like Vichy, or else alcohol or a syrup should be used as the vehicle. The latter is of course preferred. The so-called tasteless tincture does not have the same injurious action on the teeth.—*Pharmaceutical Era.*

---

A BROKEN FRIENDSHIP may be soldered up, but it will always show the break. You can impose on an enemy and it will be nothing more than he expects; but an imposition on a friend is never forgotten or forgiven.

---

MARRIED.—On Wednesday, April 25th, at the residence of the bride's parents, Miss Nannie Bell, daughter of Dr. and Mrs. J. G. Templeton, of Pittsburgh, Penna., to Walter M. Lindsay.

---

THE FIRST DISTRICT DENTAL SOCIETY will hereafter meet at the rooms of The Academy of Medicine in New York City, upon the first Monday evening of each month.

## Figures and Tables

**Figure f1:**